# A Multicentric, Open-Label, Randomized, Comparative Clinical Trial of Two Different Doses of Expanded hBM-MSCs Plus Biomaterial versus Iliac Crest Autograft, for Bone Healing in Nonunions after Long Bone Fractures: Study Protocol

**DOI:** 10.1155/2018/6025918

**Published:** 2018-02-22

**Authors:** Enrique Gómez-Barrena, Norma G. Padilla-Eguiluz, Cristina Avendaño-Solá, Concepción Payares-Herrera, Ana Velasco-Iglesias, Ferran Torres, Philippe Rosset, Florian Gebhard, Nicola Baldini, Juan C. Rubio-Suarez, Eduardo García-Rey, José Cordero-Ampuero, Javier Vaquero-Martin, Francisco Chana, Fernando Marco, Javier García-Coiradas, Pedro Caba-Dessoux, Pablo de la Cuadra, Philippe Hernigou, Charles-Henri Flouzat-Lachaniette, François Gouin, Didier Mainard, Jean Michel Laffosse, Miriam Kalbitz, Ingo Marzi, Norbert Südkamp, Ulrich Stöckle, Gabriela Ciapetti, Davide Maria Donati, Luigi Zagra, Ugo Pazzaglia, Guido Zarattini, Rodolfo Capanna, Fabio Catani

**Affiliations:** ^1^Servicio de Cirugía Ortopédica y Traumatología, Hospital de Traumatología, Hospital La Paz, IdiPAZ, Universidad Autónoma de Madrid, P° Castellana 261, 28046 Madrid, Spain; ^2^Hospital Universitario La Paz, HTR Planta 1, Despacho Médico, Universidad Autónoma de Madrid, P° Castellana 261, 28046 Madrid, Spain; ^3^Clinical Pharmacology Unit, Hospital Universitario Puerta de Hierro Majadahonda, Manuel de Falla 1, Majadahonda, 28222 Madrid, Spain; ^4^Clinical Pharmacology Unit, IIS Puerta de Hierro-Segovia de Arana, Hospital Universitario Puerta de Hierro Majadahonda, Manuel de Falla 1, Majadahonda, 28222 Madrid, Spain; ^5^IIS Puerta de Hierro-Segovia de Arana, SCRen, Manuel de Falla 1, Majadahonda, 28222 Madrid, Spain; ^6^Hospital Clínic de Barcelona, Biostatistics Unit, IDIBAPS, Carrer de Villarroel 170, 08036 Barcelona, Spain; ^7^Chirurgie Orthopédique et Traumatologique, Centre Hospitalier et Universitaire de Tours, Avenue de la République, 37170 Chambray Les Tours, France; ^8^Department of Orthopaedic Trauma, Ulm University Hospital, Albert-Einstein-Allee 23, 89081 Ulm, Germany; ^9^Dipartimento di Scienze Biomediche e Neuromotorie, Alma Mater Studiorum-Università di Bologna, SSD Fisiopatologia Ortopedica e Medicina Rigenerativa, Istituto Ortopedico Rizzoli, Via di Barbiano 1/10, 40136 Bologna, Italy; ^10^Servicio de Cirugía Ortopédica y Traumatología, Hospital Universitario La Paz, P° Castellana 26, 28046 Madrid, Spain; ^11^Servicio de Cirugía Ortopédica y Traumatología, Hospital Universitario La Princesa, Calle Diego de León 62, 28006 Madrid, Spain; ^12^Servicio de Cirugía Ortopédica y Traumatología, Hospital Universitario Gregorio Marañón, Calle del Dr. Esquerdo 46, 28007 Madrid, Spain; ^13^Servicio de Cirugía Ortopédica y Traumatología, Hospital Clínico San Carlos, Calle del Prof. Martín Lagos, s/n, 28040 Madrid, Spain; ^14^Servicio de Cirugía Ortopédica y Traumatología, Hospital Universitario 12 de Octubre, Avenida de Córdoba, s/n, 28041 Madrid, Spain; ^15^Servicio de Cirugía Ortopédica y Traumatología, Hospital Universitario Puerta de Hierro Majadahonda, Calle Manuel de Falla 1, Majadahonda, 28222 Madrid, Spain; ^16^Department of Orthopaedic Surgery, Hospital Henri Mondor, 51 Avenue du Maréchal de Lattre de Tassigny, 94010 Créteil Cedex, France; ^17^Department of Orthopaedic Surgery, CHU Nantes, 1 Place Alexis-Ricordeau, 44093 Nantes Cedex 1, France; ^18^Department of Orthopaedic Surgery, CHU Nancy, 5 rue du Morvan, 54500 Vandœuvre-lès-Nancy, France; ^19^Department of Orthopaedic Surgery, CHU Toulouse, 170 Avenue de Casselardit, 31059 Toulouse, France; ^20^Department of Trauma, Hand and Reconstructive Surgery, Universitätsklinikum Frankfurt, Universitätsklinikum-Haus 1, Theodor-Stern-Kai 7, 60590 Frankfurt am Main, Germany; ^21^Klinikfür Orthopädie und Unfallchirurgie, Universitätsklinikum Freiburg, Hugstetter Str. 55, 79106 Freiburg im Breisgau, Germany; ^22^Klinikfür Unfall- und Wiederherstellungschirurgie, Universitätsklinikum Tübingen, Schnarrenbergstraße 95, 72076 Tübingen, Germany; ^23^SSD Fisiopatologia Ortopedica e Medicina Rigenerativa, Istituto Ortopedico Rizzoli, Via di Barbiano 1/10, 40136 Bologna, Italy; ^24^Clinica Ortopedica e Traumatologica III a Prevalente Indirizzo Oncologico, Istituto Ortopedico Rizzoli, Via di Barbiano 1/10, 40136 Bologna, Italy; ^25^Chirugia dell'Anca 1, Istituto Ortopedico Galeazzi, Via Riccardo Galeazzi 4, 20161 Milano, Italy; ^26^Azienda Spedali Civili di Brescia, Piazzale Spedali Civili 1, 25123 Brescia, Italy; ^27^II Clinica Universitaria Ortopedia e Traumatologia, Azienda Ospedaliera Universitaria Pisana, Via Roma 67, 56126 Pisa, Italy; ^28^Reparto di Ortopedia e Traumatologia, Azienda Ospedaliero-Universitaria di Modena, Largo del Pozzo 71, 41125 Modena, Italy

## Abstract

ORTHOUNION is a multicentre, open, comparative, three-arm, randomized clinical trial (EudraCT number 2015-000431-32) to compare the efficacy, at one and two years, of autologous human bone marrow-derived expanded mesenchymal stromal cell (hBM-MSC) treatments versus iliac crest autograft (ICA) to enhance bone healing in patients with diaphyseal and/or metaphysodiaphyseal fracture (femur, tibia, and humerus) status of atrophic or oligotrophic nonunion (more than 9 months after the acute fracture, including recalcitrant cases after failed treatments). The primary objective is to determine if the treatment with hBM-MSCs combined with biomaterial is superior to ICA in obtaining bone healing. If confirmed, a secondary objective is set to determine if the dose of 100 × 10^6^ hBM-MSCs is noninferior to that of 200 × 10^6^ hBM-MSCs. The participants (*n* = 108) will be randomly assigned to either the experimental low dose (*n* = 36), the experimental high dose (*n* = 36), or the comparator arm (*n* = 36) using a central randomization service. The trial will be conducted in 20 clinical centres in Spain, France, Germany, and Italy under the same clinical protocol. The confirmation of superiority for the proposed ATMP in nonunions may foster the future of bone regenerative medicine in this indication. On the contrary, absence of superiority may underline its limitations in clinical use.

## 1. Introduction

Bone injuries represent an important medical problem worldwide, producing significant healthcare and societal expenditure. In Europe, complex fractures are increasing in frequency due to severe traumatic injuries, such as traffic accidents, presently with higher survivorship of polytrauma patients. While most bone injuries are capable of healing through bone regeneration by natural callus formation with standard treatments, long bone injuries may not heal, impairing the patient's life and becoming an important unmet clinical need.

Nonunions, also known as pseudarthrosis, may occur in 5% to 20% of long bone fractures that fail to heal properly, with morbidity, prolonged hospitalization, and increased expenses [1, 2]. Clinical consensus is lacking regarding the definition of nonunions [3]. Nonunions are frequently defined under the FDA guidance [4], and the absence of consolidation more than 9 months after the index fracture without evidence of progressive signs of bone healing are widely used criteria. Furthermore, nonunions are classified based on the biologic potential of bone healing [5]. Since Weber and Cech (1976), quoted by Müller et al. [6], atrophic nonunion is associated with poor vascularity and shows insufficient bone bridging to stabilize the fracture, insufficient bone biological activity in the fracture, and failure of previous treatments. These require augmentation to procure bone healing, and the current standard treatment considered as the benchmark is autologous bone grafting, obtained from the same patient at a different surgical site and transplanted to the reconstruction site.

The iliac crest holds a reservoir of spongious bone that is frequently used as a source of bone autograft, providing extracellular matrix (for osteoconduction), growth factors (modulating bone healing as per osteoinduction), and patient's cells (leading to local osteogenesis). However, major complications in 5% of the cases have been reported, including donor defect hernias, vascular injuries, sciatic nerve injury, deep infection, deep haematoma requiring transfusion, and iliac wing fracture. Minor related complications include pain at the extraction site in 5% cases, superficial haematoma or seroma or superficial infection at the extraction site in 13% cases, and ilioinguinal neuralgia in 2% cases [7]. Other limitations of iliac crest autograft (ICA) fostering research for alternatives include limited amount of available bone (particularly if previous harvesting and replacement by fibrous tissue) and limited efficacy. Although a benchmark, insufficient information about iliac crest autograft efficacy is available from the literature. When used as a control, a recent multicentric trial with 61 patients treated with autograft observed 74% radiological consolidation (45/61) by rigorous criteria (bridging in at least three of four views) at 9 months, even if only 25% of these cases were atrophic nonunions [8].

Culture-expanded autologous MSCs combined with biomaterial granules as carrier agents have been proposed as a promising technology to substitute bone autograft to augment nonunions in early feasibility studies [9, 10]. However, studies and publications vary regarding the origin of MSCs, the expansion protocol, the reproducibility and variability of the cell product, the quality of this cell product at delivery, the demographics of the treated patients, the diagnostic criteria of nonunion in the inclusion criteria, the bone healing criteria, the safety reporting, and the follow-up. Most importantly, the confirmed dose in the implanted cell product is unclear, and previous trials just declare to implant the maximum cell dose that can be generated by the available technology at the facility producing the cell expansion.

Significant preclinical work including quality assessment and standardisation of the cell product to be implanted is required before a cell product can obtain the approval for a certain trial as an investigational medicinal product (IMP) on an advanced therapy medicinal product (ATMP) under good manufacturing practices (GMP), in compliance with European regulation [11]. Besides, significant efforts in the design of a wide multicentric trial are required to confirm safety and prove efficacy, with adequate randomization against a widely accepted control, in a patient population precisely defined by specific inclusion and exclusion criteria.

As part of the EU-FP7-REBORNE project (GA 241879), the procedures of isolation, culture, and characterization of expanded human bone marrow-derived mesenchymal stromal cells (hBM-MSCs) cultured in platelet lysate (PL) were fully standardized and validated by multiple GMP production facilities in France, Italy, Germany, and Spain. The therapeutic doses obtained at release and used in the early clinical trial within the REBORNE project were 100 × 10^6^ and 200 × 10^6^ hBM-MSC cells. Cells adhered to a CE-marked granulated biphasic bioceramic and were delivered to the bone injury by open surgery after nonunion site surgical preparation. The osteogenic potential was preclinically studied both in vitro and in vivo [12, 13]. The tumorigenicity and migration of human MSCs derived from BM (tumorigenicity and biodistribution) were also preclinically studied in NOD SCID mice [14]. After validation among the GMP manufacturing centres from the four participating countries, the IMP was approved for safety and feasibility clinical trials for nonunion fractures (EudraCT number 2011-005441-13) and for avascular necrosis of femoral head (EudraCT number 2012-002010-39). No safety events related to the IMP were detected for any of these early trials. At this point, the evaluation of efficacy against the standard is still required to support the future clinical application of this technique.

MBCP+™ (Biomatlante, France) is a class III implant of wide use, CE marked (CE0123) and FDA 510(k) approved. It is a biphasic material composed of HA/*β*-TCP in a ratio of 20/80 in weight, resorbable and able to be rapidly replaced by newly formed bone [15]. The biomaterial was selected due to preclinical studies that confirmed high colonization of the biomaterial by osteogenic cells [16] and due to proven safety and efficacy of the MBCP+ alone or combined with expanded MSCs [14–16] at the selected dose [17, 18].

As a part of the EU-H2020-ORTHOUNION project (GA 733288), the global aim of this ORTHOUNION clinical trial is to overcome the major hurdle to complete the translation to clinical application. Therefore, the ORTHOUNION proposal focuses on testing the efficacy of expanded BM-hMSC in two different doses (100 and 200 × 10^6^ cells), versus iliac crest bone autograft, the currently accepted standard therapy, to biologically augment surgical treatment of long bone nonunions.

## 2. Material and Methods

### 2.1. Trial Characteristics and Design

ORTHOUNION is a multicentre, open, comparative, randomized, clinical trial with three parallel arms to evaluate the efficacy at one and two years of two doses of an ATMP against iliac bone autograft to enhance bone healing in patients with long bone nonunion (EudraCT number 2015-000431-32). A total of 108 patients will be randomly assigned to either the experimental low-dose arm, experimental high-dose arm, or the active comparator arm (ICA) with a 1 : 1 : 1 allocation using a central randomization service. The experimental low dose contains a total number of 100 × 10^6^ hBM-MSCs suspended in 5% human albumin up to a total volume of 10 mL (cell concentration of 10 × 10^6^). The experimental high dose contains a total number of 200 × 10^6^ hBM-MSCs suspended in 5% human albumin up to a total volume of 10 mL (cell concentration of 20 × 10^6^). Each experimental dose will be combined with 10 cc of the granulated biomaterial MBCP+ (Biomatlante, Nantes, France). The trial will be conducted in 20 clinical investigational sites in four European countries (France, Germany, Italy, and Spain) under the same clinical protocol and standardized surgical techniques. The cell product derived from autologous hBM-MSCs will be expanded in 4 different GMP manufacturing facilities in France (EFS Ile de France, Centre de Thérapie Cellulaire de Créteil), Germany (Institut für Klinische Transfusionsmedizin und Immungenetik Ulm Gemeinnützige GmbH (IKT Ulm), DRK-Blutspendedienst Baden-Würrtemberg–Hessen und Universitätsklinikum Ulm, Ulm), Italy (Fondazione IRCCS Ca' Granda Ospedale Maggiore Policlinico, Cell Factory “F. Calori”, Milano), and Spain (Unidad de Producción Celular UPC, Hospital Puerta de Hierro-Majadahonda, Universidad Autónoma de Madrid, Servicio de Hematología y Hemoterapia, Madrid).

### 2.2. Study Objectives

The primary objective is to determine if the combined treatment of the ATMP, *hBM-MSC + biomaterial (G2)*, is superior to the control, *iliac crest autologous graft (G1)*, to obtain radiological and clinical bone consolidation at 12 months after surgery of diaphyseal and/or metaphysodiaphyseal (femur, tibia, and humerus) atrophic or oligotrophic nonunions after a fracture (more than 9 months after the acute fracture and insufficient bone bridging to stabilize the fracture, insufficient bone biological activity in the fracture, and failure of previous treatments). The secondary objectives (SO) are the following: (SO1) to determine if the *low dose of hBM-MSC + biomaterial (G2b)* is noninferior to the *high dose of hBM-MSC + biomaterial (G2a)* to obtain radiological consolidation at 12 months after surgery of diaphyseal and/or metaphysodiaphyseal (femur, tibia, and humerus) nonunions after an unhealed fracture; (SO2) to compare the percentage of bone consolidation between the G1 and G2 treatment arms at 6 and 24 months after surgery and between the G2a and G2b treatment arms at 6, 12, and 24 months after surgery; (SO3) to compare the radiological consolidation between the G1 and G2 treatment arms and between G2a and G2b treatment arms, at baseline and 6, 12, and 24 months after surgery; (SO4) to compare pain with and without weight bearing using the Numeric Rating Scale (from 0 to 10) between the G1 and G2 treatment arms and between G2a and G2b treatment arms, at baseline and 6, 12, and 24 months after surgery; (SO5) to compare the rate of further surgical intervention at the callus site between the G1 and G2 treatment arms and between G2a and G2b treatment arms at 6, 12, and 24 months after surgery; (SO6) to compare the early and global complication rate between the G1 and G2 treatment arms and between G2a and G2b treatment arms at 6, 12, and 24 months after surgery; (SO7) to assess the safety of autologous hBM-MSCs between the G1 and G2 treatment arms at 6, 12, and 24 months after surgery; (SO8) to identify the factors associated with bone consolidation between the G1 and G2 treatment arms and between G2a and G2b treatment arms at 6, 12, and 24 months after surgery; and (SO9) to compare the physical and mental health status using the SF-36 Health Survey at baseline and 6, 12, and 24 months after surgery. Outcome measures are described in Table 1.

### 2.3. Patient Population and Evaluation

Each investigator will enroll eligible patients into the trial (after obtaining the signature of the due informed consent adapted to each national regulation) and will enter patient data into the eCRF timely throughout the study. Inclusion and exclusion criteria are shown in Table 2. During the inpatient stay, recovery, and follow-up, all participants will receive conventional treatment.

A clinical trial committee, composed by three independent expert clinicians, will review and adjudicate the primary efficacy outcomes (bone consolidation) and other collected clinical outcomes, using prespecified definitions and methods described in the Clinical Event Committee Charter. Files for adjudication will be prepared and accordingly blinded prior to adjudication.

**Figure 1:**
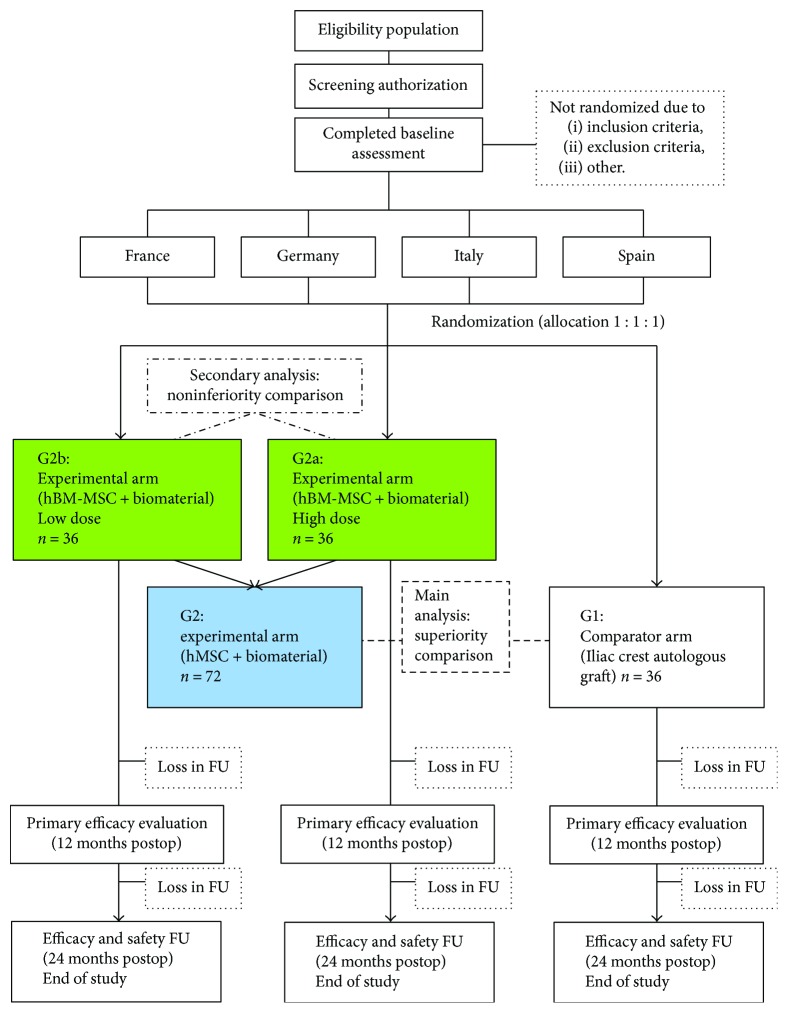
CONSORT diagram of the ORTHOUNION clinical trial.

**Figure 2:**
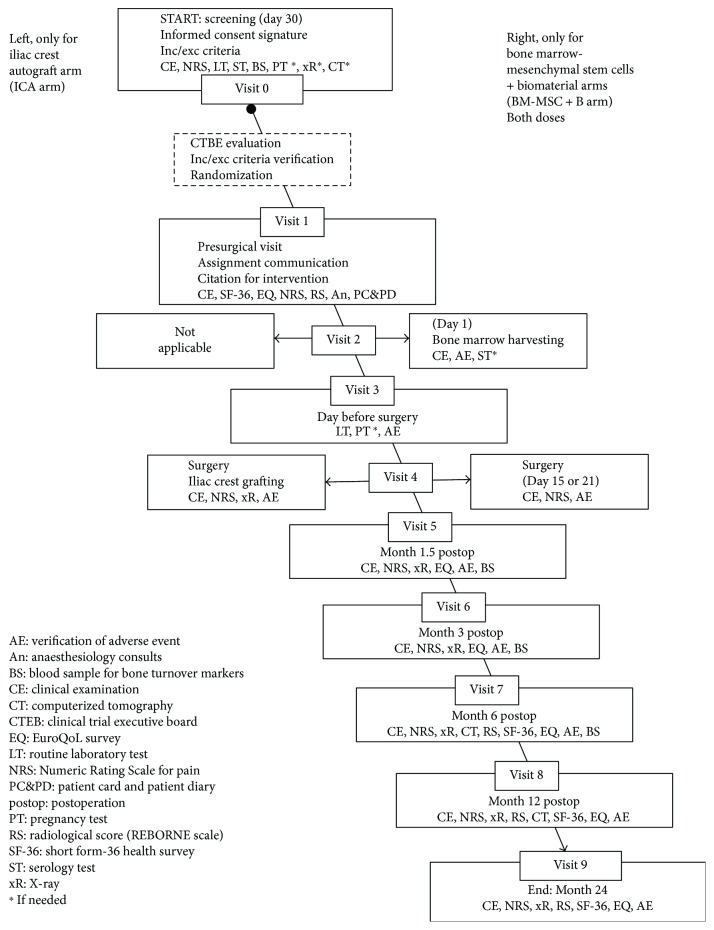
Flow diagram of the ORTHOUNION clinical trial.

Blood samples will be obtained for routine blood tests, serology tests (HIV-, syphilis, HBV, and HCV), and bone turnover marker tests (bone-specific alkaline phosphatase (BAP), C-terminal propeptide of type I procollagen (PICP), osteocalcin N-terminal fragment (Midtact-OC), carboxyterminal cross-linked telopeptide of type I collagen (beta-CTX), osteoprotegerin (OPG), receptor activator of nuclear factor NF-kappa B ligand (RANKL)). Flow diagrams of the trial are shown in Figures 1 and 2.

### 2.4. Treatments

The active comparator treatment (augmentation with spongious ICA) is a surgical procedure considered the standard of care, which requires the extraction of the needed spongious bone graft from the iliac crest at nonunion surgery. The experimental treatment (augmentation with autologous hBM-MSCs combined with the previously described CE-marked biphasic bioceramic) includes expansion of MSCs under GMP conditions in associated facilities. For both experimental arms (G2a and G2b), expanded autologous hBM-MSCs will be mixed in the surgical setting, immediately before implantation, with the previously described biomaterial called MBCP+, a class III, CE-marked, and FDA-approved biomaterial, provided by Biomatlante (Nantes, France) in a syringe ready to use.  Table 3 summarizes the study arms.

### 2.5. Surgical Interventions

The preparation of the nonunion site will be the same for both interventions. It will consist in the ablation of the necrotic bone fragments and intercalated fibrous tissue within the nonunion, followed by bone decortication to create a vascularized bed in which the cell composite or the autograft will be placed.

#### 2.5.1. Iliac Crest Grafting

The ICA will be obtained as per standard surgical technique. The appropriate incision after skin preparation will expose the anterior iliac crest, followed by blunt dissection. Once exposed, the surgeon will decide on the most appropriate trephines and dowels to be used to conservatively obtain the required spongious graft that will be preserved through the case in a separate container, as per standard of care. If insufficient autograft to fill and cover the nonunion is harvested, the incision and crest approach may be extended so the harvesting fulfils the bone graft needs. Usually, one single anterior iliac crest suffices to cover the autograft needs of about 10 cc for each case (similar volume to the implanted biomaterial in the experimental arm). In case of insufficient bone in the exposed iliac crest (due to previous harvesting or other surgical issues), the contralateral crest may be exceptionally required. By no means will the surgeon obtain insufficient bone autograft to complete the surgical requirements. The wound will be closed following muscle, fascia, and subcutaneous layer closure. A drain may be used if found necessary by the surgeon in charge of the case.

#### 2.5.2. Bone Marrow Mesenchymal Stem Cells + Biomaterial Implantation

First, the bone marrow will be harvested under loco-regional (epidural, spinal) or general anaesthesia. Bone marrow will be aspirated from the anterior or posterior iliac crests, after insertion of a bevelled needle (6 to 8 cm in length and 1.5 mm in internal diameter) into the spongy bone, and transferred into a 20 mL plastic syringe with 1 mL of heparin. At a given depth, the needle will be turned 45° to reorient the bevel during successive aspirations, so that the largest possible space is aspirated. After one full turn, the needle will be moved 1 cm toward the surface through the same insertion site, and aspirations will again be performed, with the needle always turned 45° after each aspiration. The marrow will be aspirated in small fractions (3–4 mL) to reduce the degree of dilution by peripheral blood. A total of 30–35 mL of bone marrow will be aspirated. If necessary, two or three perforations will be performed, through the same skin opening, into the iliac crest, with the perforations spaced approximately 2 cm from each other to avoid dilution by aspiration in the previous hole. All aspirates will be pooled in plastic bags containing an anticoagulant solution. The bone marrow will be then forwarded to the associated GMP facilities. The final product of expanded MSCs will be manufactured according to GMP rules using aseptic procedures and disposable sterile single-use supplies for all product contact steps. The whole process will be conducted in accordance with written procedures and each step will be recorded on batch. All manipulations involving the initial preparation of cells, cell culture, and cell packaging will be performed in clean rooms of appropriate class of air cleanliness. The batch production records for each lot require documentation and confirmation signatures that the procedures have been followed. Briefly, MSCs will be cultured and expanded using alpha MEM medium supplemented with PL [19]. Cells will be conditioned in a syringe for injection. The final product will consist of fresh, autologous bone marrow MSCs, expressing the markers CD90, CD73, CD105 and negative for CD14 and CD45 and HLA class II, with a 90% viability rate, at a dose of 100 × 10^6^ or 200 × 10^6^ cells (G2a and G2b) suspended in 5% human albumin up to 10 mL (the delivery contains two syringes of 5 mL each).

Secondly, the conduction of the surgery will be the same as for bone autograft, except for the implantation of the composite (hBM-MSCs + biomaterial). The mixture will be prepared in the operating room as per protocol [20] and placed in the previously prepared area to be grafted. Each 5 mL cell product syringe is mixed with 5 cc of biomaterial (1-2 mm MBCP+ granules) filling the intergranular pores. The final mixture of the biomaterial and cells has a pasty consistency and it will be preferably introduced within the gap of the nonunion. Circumferential use of the material will only be performed when the bone gap is filled.

In both cases, a stable fixation of the fracture will be required. The use of an osteosynthesis system may include static or locked nail, locked or unlocked bone plate with screws, and/or external fixator (including the Ilizarov system) when other means are not adequate. Furthermore, the surgical technique will be reevaluated postoperatively by the clinical trial committee, to confirm the predefined criteria of a satisfactory surgical procedure (stable fixation and adequate augmentation) to validate the analysis of the case as per protocol. These criteria include absence of major complications after the procedure (intraoperative fracture, deviation, inadequate reduction, or other surgical gestures or interventions, except fibular osteotomy if required for compression or reduction of a tibial nonunion), presence of sufficient biomaterial or graft (filled nonunion gap, presence of sufficient graft/material in the fracture compression side and not in the tension side), and sufficient fixation (detecting immediately after surgery what could be considered an unstable fixation or unstable nail or other suboptimal techniques).

## 3. Statistical Analysis

### 3.1. Sample Size

#### 3.1.1. Test of Superiority (Primary Endpoint) for G1 versus G2 Comparison

For an allocation 1 : 2 to test the percentage of bone consolidation between G1 and G2, the sample size of 36 in comparator arm (ICA) and 72 in experimental arm (hBM-MSC) achieve more than 80% power to detect superiority with a clinically meaningful margin of superiority of 20%, considering a 5% loss. Despite the uncertainties derived from the fact that no head-to-head comparisons have been tested in well-designed randomized clinical trials, literature data suggest that the control arm (G1) may have a rate of 74% in efficacy [8] and that it is reasonable to expect 20% superiority for the rate of responders in the experimental arm (G2a + G2b) [21, 22]. The significance level of the test will be established at the 2.5% one-sided alpha level (equivalent to 5% two-sided alpha level).

#### 3.1.2. Test of Noninferiority (Secondary Endpoint) for G2a and G2b Comparison

The sample size for the assessment of the noninferiority of G2b in front of the G2a arms will be based on the REBORNE scale. A total sample size of 72 participants with 36 in group G2a and 36 in group G2b will achieve more than 90% power to establish the noninferiority using a one-sided 2.5% alpha level. The margin of noninferiority is 10% on the ratio of means, assuming that the true ratio is 1.0 (i.e., G2a and G2b have the same effect). The coefficients of variation of both groups are assumed to be around 0.1349 based on internal data, with a mean of 0.113 and a SD of 0.815 [23].

All sample size calculations have been performed using the nQuery v7.0 validated software [24].

### 3.2. Plan of Analysis

Primary and secondary endpoints and the statistical plan of analysis are described in Table 1. Five subgroups for analysis are declared: manufacturing site (Spain, France S1, France S2, Germany, and Italy); anatomical site: femur, humerus, and tibia; time (months) since acute fracture; smoking habits (yes/no); and sex (female/male). For these, Fisher's exact test for categorical variables, *t*-test or one-way ANOVA for Gaussian distributed variables, Mann–Whitney test and Kruskal-Wallis for non-Gaussian continuous variables will be used.

The plan of analysis will be tested in the following two populations of analysis: (a) modified full analysis set (mFAS) that will include all patients who have received treatment; (b) per protocol population (PP) that will include those patients in the mFAS set without major protocol deviations. Procedures to account for missing or spurious data will include patients whose information on consolidation is unknown at the end of the trial and will be considered failures, irrespectively, to the drop-out reason. With regard to the radiological consolidation, the baseline value will be used to impute the missing case; in case of treatment-related missing data, the missing case is not imputed otherwise. No formal imputations will be performed for the rest of variables, and the analyses will be based on the Available Data Only (ADO) approach.

## 4. Discussion

There is a lack of multicentric, randomized clinical trials in the literature, capable of defining the value of current regenerative medicine strategies based on MSCs. Bone healing in difficult clinical settings has gathered multiple early trials with cell therapy, but the available evidence is limited.

Nonunions after long bone fractures are a challenging scenario to evaluate the efficacy of cell therapy or other competing technologies. Furthermore, the severity of the included cases may impact the results. Delayed unions of the tibia were healed by bone marrow MSCs and platelet-rich plasma [25], but obviously the severity of the cases was limited. Clinical trials on the treatment of long nonunions frequently include any type of nonunion, from hypertrophic to oligotrophic or atrophic, and the results may be impacted by the case mix. A reference multicentric trial [8] included 25% atrophic nonunions in the control arm versus 42% in the experimental arm. To solve this issue, our trial will exclude hypertrophic nonunions.

The control arm in comparative trials about bone healing is a difficult choice. Although iliac crest autograft has long been considered the standard augmentation to obtain bone healing, this control may be variable due to the patient and the surgical variability. Besides, no reference studies are available about the effectiveness of ICA to support bone healing in nonunions. Recent data can only be obtained from its use as controls in other studies [8, 26], but the variability in the obtention of ICA and the subsequent complications [7, 27, 28] foster the proposal of alternatives. We used this information to quantitatively estimate the expected healing rates with ICA, although it may vary depending on the type and location of the nonunion.

The use of cultured hBM-MSCs associated with biomaterials to heal long bone nonunions has long been reported. In 2007, Bajada et al. [29] healed a resistant tibial nonunion with 5 × 10^6^ BM-MSCs combined with calcium sulphate (CaSO_4_) in pellet form. Giannotti et al. [30] treated 8 upper limb nonunions with expanded MSCs and autologous plasma gel with CaCl_2_. Ismail et al. [31] ran a clinical trial on 10 patients with atrophic nonunions randomizing 5 patients to 15 million autologous BM-MSCs, hydroxyapatite granules, and internal fixation, with 5 controls with ICA. Larger defects were also treated by these techniques. Marcacci et al. [32] reported 4 patients with diaphyseal bone defects treated with 20 × 10^6^ expanded BM-MSCs combined with cylinders of 100% porous hydroxyapatite, with bone healing. Dilogo et al. [33] reported a case of segmental tibial defect treated with 50 × 10^6^ BM-MSCs, hydroxyapatite (HA) granules, and bone morphogenic protein 2 (BMP-2). Therefore, proofs of concept are published and feasibility is confirmed by numerous authors, although the consensus is that controlled randomized clinical trials will have to clarify definitively the effectiveness and the cost/benefit superiority of the tissue engineering approach compared to other methods of bone reconstruction [29–33].

The dose of expanded cells in previously published studies is highly variable, partly dependent on the available technology. In a previous project (FP7-REBORNE), the current IMP was developed and approved for clinical trials (EudraCT 2011-005441-13 and EudraCT 2012-002010-39), and the attained dose covered the requisites of the current trial, that is 200 million cells, expanded from 25–35 mL of bone marrow. Currently, the optimum dose to heal nonunions in long bones is unknown. Maximizing the dose to be implanted has been the most frequent strategy in many ongoing declared trials. Our high dose (200 million cells) and even the low dose (100 million cells) compare favourably with previously published bone marrow-derived cell products checked for efficacy (15 million cells used by Ismail et al. [31], 20 million cells by Labibzadeh et al. [34]).

Difficulties are encountered to define the sample size, the randomization procedure, and the study design due to the scarcity of comparable studies. A multicentric, randomized study was planned to ascertain that, having standardized the ATMP in four countries (France, Germany, Italy, and Spain), patients could receive analogous treatment from different GMP facilities in different hospitals. First, this requires agreement of the national regulators, and a voluntary harmonization procedure (VHP) was launched within the European Union [35]. Second, stratification by production centre has been planned for the randomization procedure, and this will enable to similarly assess cell production in all centres.

The sample size calculations have been based on success rates with ICA as the control on bone healing multicentric trials [8]. Scarcity of references (both for controls and technology under evaluation) is observed in the literature. Of note, no direct head-to-head comparison in a well-designed randomized clinical trial has been previously conducted with ICA and MSCs. In this scenario with uncertainties in the final success rates and in the final delta of superiority, it is also noted that very small rate changes in those assumptions may have an impact in the actual statistical power. However, clinical relevance of the potential superiority of the technique would not be clearly defined unless the success rate is clearly higher. Furthermore, other reasons for failure besides the augmentation with the ATMP need to be excluded by the clinical trial committee. Foreseen reasons of failure unrelated to the ATMP include unstable fixation or other mechanical issues, inappropriate placement of the biomaterial with cells or the bone autograft (on-lay apposition instead of the recommended in-lay application), or intraoperative complications (intraoperative fractures or other distortion). Those cases will not be evaluated by PP although ITT analysis will be performed. Justification of the noninferiority of the lower versus the higher dose of hBM-MSCs is also a potential limitation of the study. A pragmatic approach was agreed in a noninferiority margin of 10% consolidation rate based on the differences between ICA controls and technologies designed to enhance bone healing in retrospective [26] and prospective [8] studies. This difference of 10% could be considered clinically irrelevant.

Unfortunately, many published studies and clinical trials on regenerative strategies to promote bone healing fail to include sufficient detail on the treated injuries and on the implanted cell product. This precludes interpretation of the outcomes and comparison among studies. Besides, variability may not permit other investigators to replicate the study conditions and results. To drive conclusions about the outcomes of a regenerative technology is then a challenge, given the complexity of biological therapies and the wide heterogeneity of conditions where bone regeneration is required.

This phase III clinical trial intended to clarify the role of bone regenerative therapies based on autologous cell expansion for nonunion of long bones; therefore, it stands as a standard for any GMP approach in this field. The interest of this study is driven by the treated condition, where the intrinsic potential of the fracture to heal seems surpassed; the cell product, with strong preclinical quality assessment and with strict release criteria to ensure reproducibility; and the study design, where an ambitious randomized comparison is intended to clarify the efficacy of cell therapy versus iliac crest autograft and the efficacy of a higher dose of 200 × 10^6^ expanded BM-MSCs to heal nonunions versus the lower dose of 100 × 10^6^ cells that may represent less cumbersome preparation of a more sustainable cell production. The results will possibly lead to a better understanding of the clinical application of stem cell technology.

## 5. Trial Status

The ORTHOUNION EU-H2020 project started on 1 January 2017. This trial received the EudraCT number 2015-000431-32, and the clinical protocol and IMP dossier (PEI 12-061) have been submitted for regulatory evaluation through VHP (9 January 2017) and authorized (19 April 2017) for France and Spain with a procedure number VHP1031-VHP2017004. The national phase at AEMPS (Spain) and ANSM (France) also completed the approval. Furthermore, the ethical committees of Hospital Universitario Puerta de Hierro Majadahonda of Madrid, Spain, and the Comité de Protections des Personnes Est IV of Strasbourg, France, also granted the authorization of the current clinical protocol. Germany and Italy are conducting a national phase evaluation with ongoing status.

## Figures and Tables

**Table 1:** Outcome measure and statistical analysis table.

Objective	Outcome measure	Time points of evaluation	Statistical analysis
Principal objective: to determine if the combined treatment of the ATMP, hBM-MSC + biomaterial (G2), is superior to the control, iliac crest autologous graft (G1), to obtain radiological and clinical bone consolidation	Bone consolidation is considered achieved when meeting the three following criteria: (1) Radiographic bone bridging: new bone formation across the fracture site visible in 3/4 cortices, on at least 3/4 views (2) Clinical healing: pain less than 3 in a NRS (0 to 10) for pain during full weight bearing and without weight bearing [36] (3) No further surgical major intervention (nail replacement, plate replacement, or replacement of all components of the previous surgery)	At 12 months after surgery	Superiority (target delta of 20%). Percentages will be estimated using a log-binomial regression model including the treatment, manufacturing site, and baseline value of the REBORNE scale as covariates. If unexpectedly the model does not fit, the Poisson long-link distribution function with a robust variance estimator will be used instead.

SO1: to determine if the low dose of hBM-MSC + biomaterial (G2b) is noninferior to high dose of hBM-MSC + biomaterial (G2a) to obtain radiological consolidation	Score calculated in xR images from REBORNE radiological scale following the formula: REBORNE score = ∑(internal, external, anterior, and posterior cortical value^∗^)/(4 × number of evaluable cortices)	At 12 months after surgery	Noninferiority (target delta of 10%). A log-transformed data of the values will be used in a mix model for repeated measurements, only if the primary outcome reaches statistical significance. Differences between arms will be estimated through adjusted means, standard errors, and its 95% CI

SO2: to compare the percentage of bone consolidation between G1 versus G2 and G2a versus G2b treatment arms	As defined in the principal objective	(i) G1/G2: at 6 and 24 months after surgery (ii) G2a/G2b: at 6, 12, and 24 months after surgery	Fisher's exact test

SO3: to compare the radiological consolidation between G1 versus G2 and G2a versus G2b treatment arms	As defined in SO1	At baseline and 6, 12, and 24 months after surgery	*t*-test or Mann–Whitney test

SO4: to compare pain with and without weight bearing using the G1 versus G2 and G2a versus G2b treatment arms	Pain score using the Numeric Rating Scale from 0 to 10, when 0 = no pain at all and 10 = worst pain ever	At baseline and 6, 12, and 24 months after surgery	Fisher's exact test Mann–Whitney test

SO5: to compare the rate of further surgical intervention at the callus site between G1 versus G2 and G2a versus G2b treatment arms	Further surgical intervention at the callus site is considered when nail replacement, plate replacement, or replacement of all components of the previous surgery are performed.	At 6, 12, and 24 months after surgery	Fisher's exact test

SO6: to compare the early and global complication rate between G1 versus G2 and G2a versus G2b treatment arms	Early (<3 months) and global complications included the following: (i) AE related to the product application process (surgical or other, including BM or ICA harvesting) (ii) Local heterotopic ossification (iii) Local bone resorption (iv) Local osteolysis (v) Local and general infection (vi) Vascular problems (ischemia, phlebitis) (vii) Neurological problems (viii) Unexpected events (e.g., hypersensitivity, immunological, and toxic) (ix) AE related to concomitant medication (e.g., anaesthetics)	At 6, 12, and 24 months after surgery	Fisher's exact test

SO7: to assess the safety of autologous hBM-MSCs between the G1 and G2 treatment arms	Safety understood as early or global complication rates and SAE rates, related to the use of hBM-MSCs	At 6, 12, and 24 months after surgery	Fisher's exact test

SO8: to identify the factors associated with bone regeneration between G1 versus G2 and G2a versus G2b treatment arms	Association outcomes (*β*, RR)	At 6, 12, and 24 months after surgery	Log-binomial regression model and mix model for repeated measurements^∗∗^

SO9: to compare the physical and mental health status between G1 versus G2 and G2a versus G2b treatment arms	SF-36 Health Survey score	At baseline and 6, 12, and 24 months after surgery	*t*-test or Mann–Whitney test

SO: secondary objective; NRS: Numeric Rating Scale; AE: adverse event. ^∗^Cortical value: 1 point if fracture is unchanged, 2 points if callus is noncontinuous, 3 points if callus is continuous but fracture is still apparent, 4 points if callus is with same density as cortical, and 0 points if noninterpretable or nonvisible. ^∗∗^Variables of interest: manufacturing centre (nominal), anatomical site of the fracture (femur, humerus, and tibia), sex (male, female), smoking habit (yes, no), and time since acute fracture (months).

**Table 2:** ORTHOUNION clinical trial inclusion/exclusion criteria.

*Inclusion criteria*
(1) Age 18 to 65, both sexes (2) Traumatic isolated closed or open Gustilo I and II, IIIA and IIIB humerus, tibial or femur diaphyseal, or metaphysodiaphyseal fracture with a status of atrophic, oligotrophic, or normotrophic nonunion. A nonunion is defined as a fracture not healed at least 9 months after the originating trauma that meets the following criteria: (i) Insufficient bone bridging to stabilize the fracture (ii) Insufficient bone biological activity in the fracture (iii) Failure of previous treatment (including bone grafting) (3) At least 9 months from acute fracture (4) Able to understand, accept, and sign informed consent (5) Medical health coverage (6) Able to understand and accept the study constraints
*Exclusion criteria*
(1) Hypertrophic nonunions (2) Segmental bone loss requiring specific therapy (bone transport, vascularized graft, large structural allograft, megaprosthesis, etc.) (3) Unrecovered vascular or neural injury (4) Other fractures causing interference with weight bearing (5) Visceral injuries or diseases interfering with callus formation (severe cranioencephalic trauma, etc.) (6) Active infection of any location and aetiology (7) Surgical contraindication of any cause (8) Pregnancy, breast-feeding women, and women who are of childbearing age and not practicing adequate birth control. The following methods are considered adequate: (i) Combined hormonal contraception (ii) Injected hormonal contraception (iii) Implanted hormonal contraception (iv) Progesterone-only hormonal contraception associated with inhibition of ovulation (v) Placement of an intrauterine device (IUD) (vi) Placement of intrauterine hormone-releasing system (IUS) (9) Malignant tumour (past history or concurrent disease) (except carcinoma in situ or basalioma in remission) (10) History of bone harvesting on iliac crest contraindicating new iliac crest bone graft harvesting or bone marrow collection (11) Insulin-dependent diabetes (12) Any evidence (confirmed by PCR) of active infection with HIV, hepatitis B, or hepatitis C (13) Any evidence of syphilis (14) Known allergies to products involved in the production process of MSC (15) Corticoid or immunosuppressive therapy more than one week in three months prior to study inclusion (16) Autoimmune inflammatory disease (17) Current treatment by bisphosphonates not stopped three months prior to study inclusion (18) Impossibility to meet at the appointments for the follow-up (19) Participation in another therapeutic trial in the previous 3 months (20) Second nonunion in case of bilateral or multiple nonunions (only one nonunion per patient will be included in the trial)

**Table 3:** ORTHOUNION arms of treatment description.

Study arm	Treatment	Dosage	Administration
G1: ICA (active comparator arm)	Iliac crest autograft	10 cc	Local administration under surgical procedure of nonunions
G2A: high dose of BM-MSC + B (experimental arm)	Expanded bone marrow mesenchymal stem cells, plus biomaterial (MBCP+)	200 × 10^6^ cells in 10 cc of biomaterial	
G2B: low dose of BM-MSC + B (experimental arm)		100 × 10^6^ cells in 10 cc of biomaterial	

## References

[1] Giorgio Calori M., Capanna R., Colombo M. (2013). Cost effectiveness of tibial nonunion treatment: a comparison between rhBMP-7 and autologous bone graft in two Italian centres. *Injury*.

[2] Blokhuis T. J., Calori G. M., Schmidmaier G. (2013). Autograft versus BMPs for the treatment of non-unions: what is the evidence?. *Injury*.

[3] Bhandari M., Guyatt G. H., Swiontkowski M. F., Tornetta P., Sprague S., Schemitsch E. H. (2002). A lack of consensus in the assessment of fracture healing among orthopaedic surgeons. *Journal of Orthopaedic Trauma*.

[4] Food and Drug Administration (1998). *Guidance Document for Industry and CDRH Staff for the Preparation of Investigational Device Exemptions and Premarket Approval Applications for Bone Growth Stimulator Devices*.

[5] Bishop J. A., Palanca A. A., Bellino M. J., Lowenberg D. W. (2012). Assessment of compromised fracture healing. *Journal of the American Academy of Orthopaedic Surgeons*.

[6] Müller M. E., Allgöwer M., Schneider R., Willenegger H. (1991). *Manual of Internal Fixation*.

[7] Hernigou P., Desroches A., Queinnec S. (2014). Morbidity of graft harvesting versus bone marrow aspiration in cell regenerative therapy. *International Orthopaedics*.

[8] Friedlaender G. E., Perry C. R., Dean Cole J. (2001). Osteogenic protein-1 (bone morphogenetic protein-7) in the treatment of tibial nonunions. *The Journal of Bone and Joint Surgery-American*.

[9] Gómez-Barrena E., Rosset P., Müller I. (2011). Bone regeneration: stem cell therapies and clinical studies in orthopaedics and traumatology. *Journal of Cellular and Molecular Medicine*.

[10] Gómez-Barrena E., Rosset P., Lozano D., Stanovici J., Ermthaller C., Gerbhard F. (2015). Bone fracture healing: cell therapy in delayed unions and nonunions. *Bone*.

[11] European Medicines Agency Scientific Guidelines for Human Medicinal Products, Clinical Efficacy and Safety Guidelines, General Guidelines. http://www.ema.europa.eu/ema/index.jsp?curl=pages/regulation/general/general_content_000366.jsp&mid=WC0b01ac0580032ec4.

[12] Veronesi E., Murgia A., Caselli A. (2014). Transportation conditions for prompt use of ex vivo expanded and freshly harvested clinical-grade bone marrow mesenchymal stromal/stem cells for bone regeneration. *Tissue Engineering Part C: Methods*.

[13] Brennan M. A., Renaud A., Amiaud J. (2014). Pre-clinical studies of bone regeneration with human bone marrow stromal cells and biphasic calcium phosphate. *Stem Cell Research & Therapy*.

[14] Rapp A. E., Bindl R., Recknagel S. (2016). Fracture Healing Is Delayed in Immunodeficient NOD/scid‑IL2Rγ_c_^null^ Mice. *PLoS One*.

[15] Bassi G., Guilloton F., Menard C. (2015). Effects of a ceramic biomaterial on immune modulatory properties and differentiation potential of human mesenchymal stromal cells of different origin. *Tissue Engineering Part A*.

[16] Miramond T., Corre P., Borget P. (2014). Osteoinduction of biphasic calcium phosphate scaffolds in a nude mouse model. *Journal of Biomaterials Applications*.

[17] Gamblin A. L., Brennan M. A., Renaud A. (2014). Bone tissue formation with human mesenchymal stem cells and biphasic calcium phosphate ceramics: the local implication of osteoclasts and macrophages. *Biomaterials*.

[18] Mebarki M., Coquelin L., Layrolle P. (2017). Enhanced human bone marrow mesenchymal stromal cell adhesion on scaffolds promotes cell survival and bone formation. *Acta Biomaterialia*.

[19] Fekete N., Gadelorge M., Fürst D. (2012). Platelet lysate from whole blood-derived pooled platelet concentrates and apheresis-derived platelet concentrates for the isolation and expansion of human bone marrow mesenchymal stromal cells: production process, content and identification of active components. *Cytotherapy*.

[20] Léotot J., Lebouvier A., Hernigou P., Bierling P., Rouard H., Chevallier N. (2015). Bone-forming capacity and biodistribution of bone marrow-derived stromal cells directly loaded into scaffolds: a novel and easy approach for clinical application of bone regeneration. *Cell Transplantation*.

[21] Machin D., Campbell M. J. (1987). *Statistical Tables for Design of Clinical Trials*.

[22] Fleiss J. L., Tytun A., Ury H. K. (1980). A simple approximation for calculating sample sizes for comparing independent proportions. *Biometrics*.

[23] Hauschke D., Kieser M., Diletti E., Burke M. (1999). Sample size determination for proving equivalence based on the ratio of two means for normally distributed data. *Statistics in Medicine*.

[24] Elashoff J. D. (2007). *nQuery Version 7.0 Advisor User’s Guide*.

[25] Liebergall M., Schroeder J., Mosheiff R. (2013). Stem cell–based therapy for prevention of delayed fracture union: a randomized and prospective preliminary study. *Molecular Therapy*.

[26] Tressler M. A., Richards J. E., Sofianos D., Comrie F. K., Kregor P. J., Obremskey W. T. (2011). Bone morphogenetic protein-2 compared to autologous iliac crest bone graft in the treatment of long bone nonunion. *Orthopedics*.

[27] Goulet J. A., Senunas L. E., DeSilva G. L., Greenfield M. L. V. H. (1997). Autogenous iliac crest bone graft: Complications and functional assessment. *Clinical Orthopaedics and Related Research*.

[28] Sen M. K., Miclau T. (2007). Autologous iliac crest bone graft: should it still be the gold standard for treating nonunions?. *Injury*.

[29] Bajada S., Harrison P. E., Ashton B. A., Cassar-Pullicino V. N., Ashammakhi N., Richardson J. B. (2007). Successful treatment of refractory tibial nonunion using calcium sulphate and bone marrow stromal cell implantation. *Journal of Bone and Joint Surgery - British Volume*.

[30] Giannotti S., Bottai V., Ghilardi M. (2013). Treatment of pseudoarthrosis of the upper limb using expanded mesenchymal stem cells: a pilot study. *European Review for Medical and Pharmacological Sciences*.

[31] Ismail H. D., Phedy P., Kholinne E. (2016). Mesenchymal stem cell implantation in atrophic nonunion of the long bones: a translational study. *Bone & Joint Research*.

[32] Marcacci M., Kon E., Moukhachev V. (2007). Stem cells associated with macroporous bioceramics for long bone repair: 6- to 7-year outcome of a pilot clinical study. *Tissue Engineering*.

[33] Dilogo I. H., Kamal A. F., Gunawan B., Rawung R. V. (2015). Autologous mesenchymal stem cell (MSCs) transplantation for critical-sized bone defect following a wide excision of osteofibrous dysplasia. *International Journal of Surgery Case Reports*.

[34] Labibzadeh N., Emadedin M., Fazeli R. (2016). Mesenchymal stromal cells implantation in combination with platelet lysate product is safe for reconstruction of human long bone nonunion. *Cell Journal*.

[35] Gomez-Barrena E., Sola C. A., Bunu C. P. (2014). Regulatory authorities and orthopaedic clinical trials on expanded mesenchymal stem cells. *International Orthopaedics*.

[36] Jensen M. P., Chen C., Brugger A. M. (2003). Interpretation of visual analog scale ratings and change scores: a reanalysis of two clinical trials of postoperative pain. *The Journal of Pain*.

